# Visual quality analysis using the Chinese Catquest-9SF scale following different spherical aberration IOL implantation

**DOI:** 10.3389/fpubh.2022.1029002

**Published:** 2022-11-03

**Authors:** Du Wei, Lou Wei, Hua Yanjun, Xu Zequan, Jin Lei, Wu Qiang

**Affiliations:** ^1^Department of Ophthalmology, The Affiliated Sixth People's Hospital, Shanghai Jiao Tong University, Shanghai, China; ^2^Department of Ophthalmology, The Affiliated Xuzhou Municipal Hospital of Xuzhou Medical University, Xuzhou First People's Hospital, Xuzhou Eye Disease Prevention and Treatment Institute, Xuzhou, China

**Keywords:** visual quality, the Chinese Catquest-9SF scale, spherical aberration, aspheric IOL, contrast sensitivity

## Abstract

**Purpose:**

Based on the Chinese version of the Catquest-9SF scale, the contrast sensitivity meter and wavefront aberrometer were used to evaluate the visual quality of cataract patients implanted with different spherical aberrations IOL.

**Design:**

Retrospective Observational Study.

**Methods:**

Patients who had the lens implantation in our department from January 2020 to December 2021 were enrolled. All patients underwent uncorrected visual acuity, best corrected visual acuity and slit lamp microscope, high-order aberrations and contrast sensitivity test. The KR-1W wavefront analyzer (Topcon Medical System, Tokyo, Japan) was used to measure wavefront aberrations post-operation. The Chinese Catquest-9SF scale was used to score the postoperative visual satisfaction of the patients.

**Results:**

145 patients were screened according to the exclusion criteria, including 51 patients in the zero aspherical IOL (SOFTEC HD) group, 42 patients in the negative aspherical IOL (ZCB00) group, and a total of 52 patients in the spherical IOL (HQ-201HEP) group. The score was the highest in the zero spherical aberration group, followed by the negative spherical aberration group with the lowest scores in the spherical IOL group. Higher-order aberrations are relatively low in eyes implanted with the zero spherical aberration group. Contrast sensitivity with spherical lenses under glare-free and glare conditions was lower than those with aspheric lenses, and at higher frequencies the zero-aberration aspheric lens performed the best.

**Conclusion:**

The Chinese Catquest-9SF scale provides an indication of visual quality after aspheric IOL implantation.

## Highlights

- There is disagreement on the extent to which reduced spherical aberration can optimize the patient's postoperative visual satisfaction.
- The present study uses the Chinese version of the Catquest-9SF scale to compare contrast sensitivity and ocular aberrations to evaluate the visual quality of cataract patients after surgery with three different IOL.
- Contrast sensitivity with spherical lenses under glare-free and glare conditions was lower than with aspheric lenses, and at higher frequencies the zero-aberration aspheric lens performed best.
- In regard to the evaluation of the visual quality. The Chinese Catquest-9SF scale provides an indication of visual quality after aspheric IOL implantation.

## Introduction

The implantation of an aspheric IOL with modified spherical aberration can reduce the postoperative global aberration to some extent and increase the contrast sensitivity in dark and light environment, thereby improving postoperative visual quality (1–5). There is disagreement on the extent to which reduced spherical aberration can optimize the patient's postoperative visual satisfaction. Some researchers assert that zero residual spherical aberration can improve the postoperative contrast sensitivity of patients, thus improving postoperative vision quality, while others point out that limited postoperative depth of focus means that some spherical aberration remains after the surgery (6–11). Previous research has used higher-order aberrations or contrast sensitivity to evaluate the patient's postoperative visual quality, but these are not complete indicators of patients' postoperative overall visual satisfaction. In recent years, vision-related quality of life scales have been widely used in clinics for subjective evaluation. A variety of scales are applied in clinical practice, including the VF-14 scale, Activities of Daily Vision Scale questionnaire (ADVS), and Vision-Related Quality of Life and Visual Function questionnaire (NEI-VFQ25) (12–18). However, the above scales and questionnaires were not validated in the Chinese population, so their applicability in this population is unknown. The Catquest-9SF scale, for subjective evaluation of postoperative visual quality, is derived from Catquest quantitative performance. It has been translated into various languages, which have been accepted by the International Consortium for Health Outcomes Measurement (ICHOM) to assess the recovery of visual function after cataract surgery (19–23). Our previous research has shown that the Chinese version of the Catquest-9SF scale has good one-dimensionality and effectiveness (24).

Therefore, the present study uses the Chinese version of the Catquest-9SF scale to compare contrast sensitivity and ocular aberrations to evaluate the visual quality of cataract patients with three different IOL.

## Materials and methods

### Study design

This is a retrospective study. Data were collected between January 2020 to December 2021. The trial was conducted in accordance with the principles of the Declaration of Helsinki.

The study population consisted of 145 subjects who underwent cataract phacoemulsification and IOL implantation in our department. All patients underwent tests of uncorrected visual acuity, best corrected visual acuity, higher-order aberrations, contrast sensitivity, and slit lamp microscopy after cataract surgery. Patients were included if they fulfilled the following criteria: (1) simple phacoemulsification + IOL implantation; (2) no intraoperative or postoperative eye complications; (3) no irregular corneal astigmatism; (4) normal pupil responses and dilated pupil diameter >5 mm. Patients meeting any of the following criteria were excluded: (1) Other diseases of the eye, such as corneal disease, glaucoma, uveitis, retinopathy, or high refractive error; (2) serious cardiopulmonary diseases or diabetes; (3) fundus lesions that affect visual quality, such as macular epiretinal membranes or age-related macular degeneration; (4) severe postoperative complication; (5) significant tilt or eccentricity of IOL after surgery.

We compared the postoperative visual quality of a zero spherical aberration aspherical IOL (Softec HD), a negative aspherical IOL (ZCB00), and a spherical IOL (HQ-201HEP). The Softec HD™ posterior chamber IOL (Lenstec Inc., St. Petersburg, FL, USA) is an ultraviolet (UV)-absorbing, single-piece modified “C” loop IOL (IOL) with a symmetrical anterior and posterior surface aspheric design (zero aberration). It is manufactured completely from a medical-grade copolymer of hydrophilic acrylic hydroxyethyl methacrylate (HEMA, 26% water content) and a polymerizable UV blocker. The overall length of the lens is 12.0 mm. The 5.5-mm-long lens optic has a 360° square edge design, designed for placement in the capsular bag. This lens is offered in power options in 0.25-D steps across the +18 to +25 D range, allowing more precise power correction. Tecnis ZCB00 is a one-piece 6.0 mm biconvex hydrophobic acrylic lens with anterior aspheric surface that resulted in a negative SA of −0.27 μm and frosted continuous 360° posterior square edge. This lens is offered in power options in 0.5-D steps across the +5 to +34 D range. It is an UV-blocking hydrophobic acrylic, single-piece modified “C” loop IOL (IOL) with a ProTEC frosted, continuous 360° posterior square edge. The HQ-201HEP (HexaVision, Inc.) is a conventional spheric IOL with a positive SA (Table 1).

**Table 1:** Comparison of three IOL.

**IOL type**	**SOFTEC HD**	**ZCB00**	**HQ-201 HEP**
IOL spherical Aberration	0 μm	−0.27 μm	>0 μm
IOL design	Aspherical IOL	Aspherical IOL	Spherical IOL
IOL material	Hydrophilic-PMMA	Hydrophobic-PMMA	Hydrophilic-PMMA
IOL diameter (mm)	12	13	12.5
Pupil diameter (mm)	5.75	6.0	6.0
Constant-A	118	119.3	118.2
IOL refractive power (D; Mean, SD)	+15.25 D~24.7 D	+5.0 D ~+34.0 D	+4.0 D~ +34.0 D

D, diopters.

### Outcome measurements

Uncorrected distance visual quality (UDVA) and best corrected visual acuity (BCVA) were accessed using a log MAR chart. BCVA was measured following refraction. Prior to acuity measurement, an autorefractor (NIDEK AOS-1500, Japan) was used to measure refractive error. Patients were required to blink to exclude the effect of tear film instability and then measurements were taken following the instrument specifications. These objective data were used as a basis for subjective refraction, which was worn to record monocular best corrected visual acuity.

The CSV-1000 contrast sensitivity test (Vector Vision, Ohio, USA) was used to measure monocular contrast sensitivities. Mean luminance of the display was 85 cd/m^2^ and was constant during the test, minimizing pupil size changes. The patient adjusted to dark room light levels for 5 mins, seated at a distance of 2.5 m from the display, and contrast sensitivity values of each eye were recorded with and without glare (45 cd/m^2^).

The KR-1W wavefront analyzer (Topcon Medical System, Tokyo, Japan) was used to measure wavefront aberrations post-surgery. The Chinese Catquest-9SF scale score was used to measure visual quality (Table 2).

**Table 2:** Version of the Chinese Catquest-9SF.

**Item**	**Chinese Catquest-9SF**
Q1	Vision difficulty in everyday life
Q2	Vision satisfaction in general
Q3	Reading text in the newspaper
Q4	Recognizing the faces of people around you
Q5	Seeing prices of goods when shopping, or descriptions on medicine bottles or bank receipts, electricity bill, water account, etc.
Q6	Seeing to walk on uneven ground.
Q7	Reading text on TV or in movie or on advertising board
Q8	Seeing to do delicate work (needlework, handiwork, carpentry, etc.)
Q9	Seeing to carry on an activity/hobby you are interested in, such as photography, calligraphy, Mah-jongg playing

The Chinese Catquest-9SF questionnaire contains 9 questions. The response options are as follows: 1 = very great difficulty; 2 = great difficulty; 3 = some difficulty; 4 = no difficulty; and 5 = cannot decide. There is also one global question about general satisfaction. The response options are as follows: 1 = very dissatisfied; 2 = rather dissatisfied; 3 = fairly satisfied; 4 = very satisfied; and 5 = cannot decide. The response category “cannot decide” is treated as missing data in the analysis.

### Statistical analysis

The data were processed and analyzed statistically using SPSS version 23.0 (IBM Corporation, Chicago, IL, USA). The sample size was chosen to achieve a statistical power of 80% for group comparisons at a 5% significance level. The age, Chinese Catquest-9SF scores, contrast sensitivity, higher-order aberrations and visual acuity measurements of patients with each of the three types of aspheric IOL were expressed in the form of mean ± standard deviation. The correlation between the Chinese Catquest-9SF scale score and visual acuity was expressed by a simple linear regression equation. Contrast sensitivity, higher-order aberrations and scale scores were compared between the three lenses using non-parametric tests (Kruskal-Wallis test). The range of the best spherical aberration was confirmed according to the score result of the scale using non-parametric test. *P* < 0.05 was considered significant.

## Results

### Patient assignment and baseline characteristics

A total of 145 eyes of 145 people were included in the study (56 males, 89 females). Fifty-one eyes were implanted with SOFTEC HD IOL, 42 eyes were implanted with ZCB00, and 52 eyes were implanted with HQ-201HEP. Mean age of patients with each type of lens was 73.31 (±8.74), 72.64 (±8.95) and 74.13 (±8.20) years, respectively. No significant difference in age was found between the three groups (*P* > 0.05) (Table 3).

**Table 3:** Baseline patient characteristics.

**Characteristics**	**Total group**	**SOFTEC HD group**	**ZCB00 group**	**HQ-201HEP**
Eye	145	51	42	52
Age	73.41 ± 8.58	73.31 ± 8.74	72.64 ± 8.95	74.13 ± 8.20
HOA (μm)	0.31 ± 0.24	0.24 ± 0.12	0.26 ± 0.19	0.42 ± 0.31
TOA (μm)	0.21 ± 0.13	0.21 ± 0.12	0.24 ± 0.18	0.19 ± 0.08
FOA (μm)	0.12 ± 0.08	0.11 ± 0.48	0.09 ± 0.09	0.16 ± 0.08
CA (μm)	0.12 ± 0.09	0.12 ± 0.07	0.14 ± 0.12	0.11 ± 0.06
SA (μm)	0.10 ± 0.13	0.08 ± 0.04	0.02 ± 0.06	0.18 ± 0.17
Score	32.51 ± 3.10	34.49 ± 1.46	32.93 ± 2.23	30.23 ± 3.42
UCVA	0.75 ± 0.22	0.81 ± 0.21	0.75 ± 0.21	0.68 ± 0.23
BCVA	0.88 ± 0.21	0.96 ± 0.21	0.89 ± 0.18	0.79 ± 0.20

HOA, higher-order aberrations; TOA, third-order aberrations; FOA, fourth-order aberrations; CA, coma; SA, spherical aberration; UCVA and BCVA, uncorrected and best corrected visual acuity, respectively.

### Correlation between Chinese Catquest-9SF score and visual acuity

**Figure 1:**
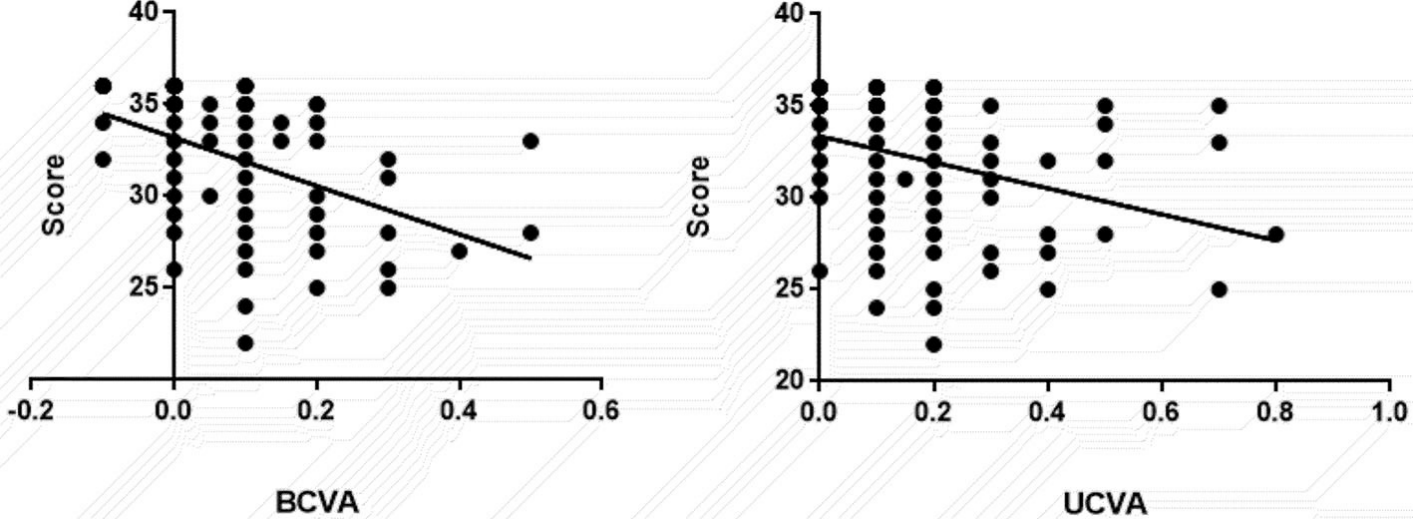
Correlation between uncorrected visual acuity (UCVA) and best corrected visual acuity (BCVA) and scale scores.

Figure 1 shows that UCVA and BCVA are significantly negatively correlated with the Chinese Catquest-9SF scale score.

### Comparison of contrast sensitivity between groups

Contrast sensitivity at the full range of spatial frequencies was significantly lower in the spherical group (HQ-201HEP) than in the aspheric groups (SOFTEC HD, ZCB00; *P* < 0.05). Within the aspheric group in medium, non-glare and glare lighting conditions, contrast sensitivity at low spatial frequencies [3 cycles per degree (c/d) and 6 c/d] was higher in the negative spherical aberration aspheric IOL group (ZCB00) than the zero spherical aberration aspheric IOL group (SOFTEC HD), while at higher spatial frequencies (12 c/d and 18 c/d) the converse was true (Tables 4, 5).

**Table 4:** Contrast sensitivity in non-glare state (mean ± SD).

**Spatial frequency (c/d)**	**SOFTEC HD**	**ZCB00**	**HQ**
3	1.31 ± 0.43	1.34 ± 0.40	1.17 ± 0.50
6	1.54 ± 0.40	1.60 ± 0.40	1.40 ± 0.47
12	1.10 ± 0.51	1.08 ± 0.48	0.95 ± 0.52
18	0.65 ± 0.46	0.61 ± 0.49	0.52 ± 0.49

**Table 5:** Contrast sensitivity in glare state (mean ± SD).

**Spatial frequency (c/d)**	**SOFTEC HD**	**ZCB00**	**HQ**
3	1.40 ± 0.28	1.40 ± 0.33	0.98 ± 0.48
6	1.54 ± 0.38	1.57 ± 0.36	1.27 ± 1.32
12	1.22 ± 0.43	1.19 ± 0.43	0.78 ± 0.46
18	0.67 ± 0.39	0.66 ± 0.44	0.35 ± 0.45

### Comparison of different aberrations between groups

The overall higher-order aberration was significantly lower in the zero spherical aberration aspheric IOL group (SOFTEC HD; 0.24 ± 0.1 μm) and in the negative spherical aberration aspheric IOL group (ZCB00; 0.26 ± 0.19 μm) than in the spheric IOL group (HQ-201HEP; 0.42 ± 0.31 μm) (*P* = 0.0078 and 0.0151, respectively). No significant difference was found between the three groups in third-order aberration or coma. The fourth-order aberration was significantly lower in the zero spherical aberration aspheric IOL group (SOFTEC HD; 0.11 ± 0.48 μm) and in the negative spherical aberration aspheric IOL group (ZCB00; 0.09 ± 0.09 μm) than in the HQ-201HEP group (0.16 ± 0.08 μm; *P* = 0.0447 and 0.0001, respectively), and significantly lower in the negative spherical aberration aspheric IOL group (ZCB00; 0.09 ± 0.09 μm) than in the zero spherical aberration aspheric IOL group (SOFTEC HD; 0.11 ± 0.48 μm; *P* = 0.0321). Overall spherical aberration was significantly lower in the zero spherical aberration aspheric IOL group (SOFTEC HD; 0.08 ± 0.04 μm) and the negative spherical aberration aspheric IOL group (ZCB00; 0.02 ± 0.06 μm) than in the spheric IOL group (HQ-201HEP; 0.18 ± 0.17 μm; *P* = 0.0146 and < 0.0001 respectively), and was significantly lower in the negative spherical aberration aspheric IOL group (ZCB00; 0.02 ± 0.06 μm) than in the zero spherical aberration aspheric IOL group (SOFTEC HD; 0.08 ± 0.04 μm) (Table 6).

**Table 6:** Comparison of higher-order aberrations, fourth-order aberrations and spherical aberrations of three intraocular lenses (mean ± SD).

**Groups**	**SOFTEC HD**	**ZCB00**	**HQ**	***P***
High-order	0.24 ± 0.12	0.26 ± 0.19	–	>0.9999
aberration (μm)	0.24 ± 0.12	–	0.42 ± 0.31	0.0078^*^
	–	0.26 ± 0.19	0.42 ± 0.31	0.0151^*^
Fourth-order	0.11 ± 0.48	0.09 ± 0.09	–	0.0321^*^
aberration (μm)	0.11 ± 0.48	–	0.16 ± 0.08	0.0447^*^
		0.09 ± 0.09	0.16 ± 0.08	< 0.0001^*^
Spherical	0.08 ± 0.04	0.02 ± 0.06	–	< 0.0001^*^
aberration (μm)	0.08 ± 0.04	–	0.18 ± 0.17	0.0146^*^
	–	0.02 ± 0.06	0.18 ± 0.17	< 0.0001^*^

* stands for *P* < 0.05.

### Comparison of scale scores between groups

**Figure 2:**
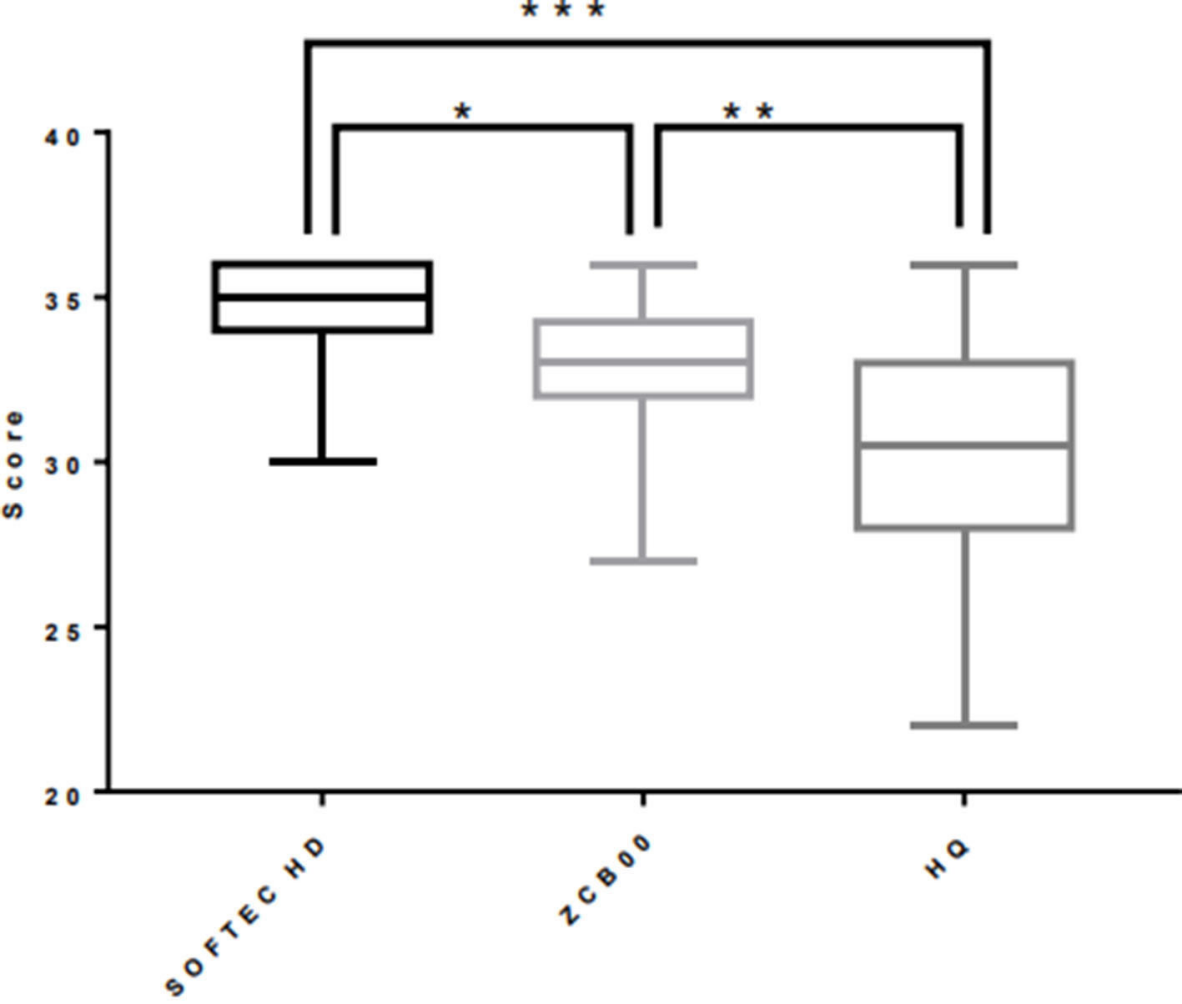
Comparison of scale scores between groups. *P = 0.0036; **P = 0.0018; ***P < 0.0001.

The scale scores were significantly different between all three groups. The scores of the zero spherical aberration aspheric IOL group (SOFTEC HD; 34.49 ± 1.46) and the negative spherical aberration aspheric IOL group (ZCB00; 32.93 ± 2.23) were higher than the spheric IOL group (HQ-201HEP; 30.23 ± 3.42) (*P* < 0.0001 and *P* = 0.0018, respectively) and the score in the zero spherical aberration aspheric IOL group (SOFTCE HD) was higher than that of the negative spherical aberration aspheric IOL group (ZCB00; *P* = 0.0036) (Figure 2).

### The range of the best spherical aberration based on the score of the scale

**Figure 3:**
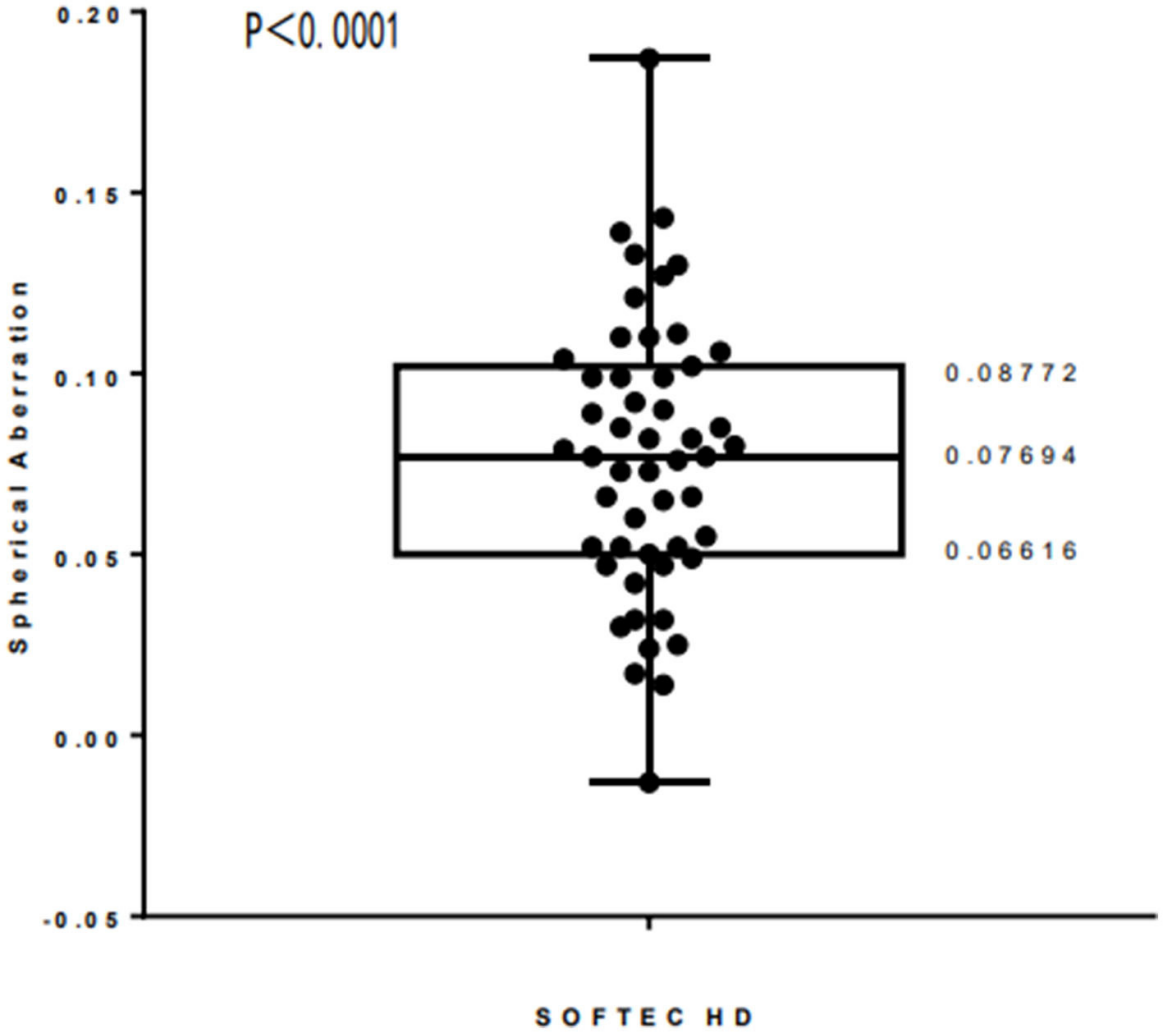
The range of the best spherical aberration based on the scale score results.

One-sample rank sum test was performed on the overall spherical aberration after surgery in the zero spherical aberration aspheric IOL group. According to the score of the scale using non-parametric test, the 95% confidence interval of the overall spherical aberration after surgery was between 0.06616 and 0.08772 μm, *P* < 0.0001 (Figure 3). So, the range of the best spherical aberration is 0.06616–0.08772 μm.

## Discussion

The use of aspherical rather than spherical IOL may reduce spherical aberrations and increase contrast sensitivity, but the impact of this improvement on overall visual quality is not well understood. Most previous studies have evaluated visual quality in postoperative patients using a single subjective or objective measure (25–29). Our research combined the Chinese Catquest-9SF scale score with residual spherical aberration of the eye after surgery to evaluate postoperative visual quality from subjective and objective viewpoints. The results of our study revealed that when the residual spherical aberration of the eye was 0.06616–0.08772 μm, the postoperative visual quality was excellent.

We compared the postoperative visual quality of a zero spherical aberration aspherical IOL, a negative aspherical IOL, and a spherical IOL. Consistent with previous studies (30, 31), contrast sensitivity and postoperative scale scores are higher and spherical aberration lower with aspheric than with spherical IOLs. Different scholars have different views about the extent to which residual spherical aberration can affect postoperative visual satisfaction. Garcin et al. (32) used the optical quality analysis system (OQAS) visual quality assessment system to study the postoperative visual quality with zero spherical aberration IOL. The authors reported that the best corrected visual acuity and contrast sensitivity were significantly improved after surgery. Zhang et al. demonstrated that (33) an aspheric IOL with a low negative or zero primary spherical aberration is recommended for cataract patients with high myopia. Hervella et al. (34) found that negative values of spherical aberration extend the depth of focus in different ways depending on each patient. Tzamalis et al. (35) revealed that Bioline yellow IOL indicated lower contrast sensitivity under mesopic conditions when glare was applied but resulted in less trefoil aberrations after uneventful cataract surgery. Although the above assessments are based on objective data comparing postoperative residual global aberration and visual quality, they all have some limitations.

Many related visual function scales exist, including the VA-14, ADVS and the NEI-VFQ25 (12–18) but their content is complex. In 1995, the Swedish National Cataract Registry Center used the Catquest questionnaire to collect data on visual function in cataract patients before and after surgery (22). The questionnaire has since been subjected to Rasch analysis, reduced from 12 to nine questions, and named the Catquest-9SF scale (19). The Catquest-9SF scale has been translated into different languages and has been endorsed by the ICHOM agency for the evaluation of visual function after cataract surgery. Recently, Xu et al. (24) applied Rasch model analysis to develop and verify the Chinese version of the Catquest-9SF scale. In the present study, the Chinese version of the Catquest-9SF was used to analyze scores among patients with three different IOL types including two aspheric lenses with different levels of spherical aberration and one spherical lens. Our research revealed that the score was the highest in the zero spherical aberration group, followed by the negative spherical aberration IOL group, and the lowest scores in the spherical IOL group. Linear regression analysis showed a significant positive correlation between the scale score and both uncorrected and best corrected visual acuities, in broad agreement with previous work (21, 25). In addition, our study revealed a significant difference between the two aspherical IOL scores. It is probable that difference levels of aberration with these two lens types in our study led to levels of difficulty in understanding the questions, which leads to differences in scores.

We found that contrast sensitivity with spherical lenses under glare-free and glare conditions was lower than with aspheric lenses, and at higher frequencies the zero-aberration aspheric lens performed the best. Our research shows that higher-order aberrations are relatively low in eyes implanted with zero spherical aberration aspheric IOL. For total spherical aberration, the negative spherical aberration aspheric IOL group is lower than the zero spherical aberration aspheric IOL group. Caporossi et al. (36) implanted one of three types of aspherical IOL or one type of spherical IOL in 250 eyes of 125 patients and demonstrated that the mean total spherical aberration was statistically lower in dominant eyes with aspheric IOLs compared with eyes with spherical IOLs. Lee et al. (37) randomly implanted three types of IOL with different spheric aberrations, and found no statistically significant difference between the best corrected visual acuity or refractive errors, and higher-order aberrations of zero spherical aberration aspheric IOL group after surgery were relatively lower than the other type of aspheric IOL. Zhao et al. (38) concluded that the aspherical acrylic IOL can reduce the higher-order aberrations (especially the spherical aberration) and increase the contrast sensitivity to improve the visual performance. Our research results are consistent with these findings.

Third-order aberration and coma mainly reflect the tilt and eccentricity of the IOL. In the present study, no significant difference in these types of aberration was found between the three IOL, suggesting that any tilt or eccentricity was similar in the three IOL.

Numerous studies have shown that higher-order aberrations on the anterior surface of the cornea of cataract patients vary greatly among individuals, and that corneal spherical aberration has the greatest impact on postoperative visual quality. This type of aberration is rotationally symmetric, making it the only high-order aberration that can be corrected using IOLs. Inter-individual differences in corneal spherical aberration mean that to optimize postoperative visual acuity it is vital to implant an aspheric IOL based on corneal spherical aberration. Studies on non-Chinese populations indicate that the average corneal spherical aberration of the human eye is 0.27 μm (39, 40) whereas the average in Chinese is 0.312 (±0.114) μm (41). Therefore, implantation of aspherical IOL based on non-Chinese corneal spherical aberration data may produce errors. Notably, personalized implantation of aspherical IOL should be based on the different ethnic groups.

Studies by Zhang et al. (42) have shown that higher-order aberrations of the corneal anterior surface may also depend on age. Young people have greater corneal spherical aberrations and less coma and with increasing age, the coma and trefoil gradually increase and corneal spherical aberration gradually decreases. The present study included an elderly population with total spherical aberration of 0.08 (± 0.04) μm after implantation of aspherical IOL. This study used the Chinese Catquest-9SF scale to analyze the correlation between visual quality and the spherical aberration of the eye after implantation of two types of aspheric IOL with different spherical aberration values and one type of spherical IOL. The post-surgery scale score was highest with zero-spherical aberration lenses. The range of the best spherical aberration is confirmed according to the score result of the scale using non-parametric test. Therefore, we believe that the total aberration after aspheric IOL implantation is within this range, and the postoperative visual quality is better.

This study has some limitations. Firstly, recent study (41) has shown that there is a certain correlation between the depth of focus and the higher-order aberrations, so how to balance the relationship between the two factors to obtain the best visual quality needs to be investigated in our further research. Secondly, the sample size was limited. Further research with larger sample size is desirable to studying the visual quality of various intraocular lenses including multifocal intraocular lens implantation. In addition, the total postoperative global aberration measured in this study included only the anterior corneal and IOL spherical aberrations, and not the posterior corneal spherical aberration, so further studies should take into account the other higher-order aberrations that may affect visual quality.

To conclude, the Chinese Catquest-9SF scale provides an indication of visual quality after aspheric IOL implantation.

## Data availability statement

The original contributions presented in the study are included in the article/supplementary material, further inquiries can be directed to the corresponding author.

## Ethics statement

The studies involving human participants were reviewed and approved by Ethics Committee of Shanghai Sixth People's Hospital. The patients/participants provided their written informed consent to participate in this study.

## Author contributions

Conceptualization: DW and WQ. Methodology: DW, HY, and LW. Formal analysis and investigation: DW, LW, and XZ. Writing—original draft preparation: DW. Writing—review and editing: DW and HY. Resources: WQ and JL. Supervision: WQ. All authors contributed to the article and approved the submitted version.

## Conflict of interest

The authors declare that the research was conducted in the absence of any commercial or financial relationships that could be construed as a potential conflict of interest.

## Publisher's note

All claims expressed in this article are solely those of the authors and do not necessarily represent those of their affiliated organizations, or those of the publisher, the editors and the reviewers. Any product that may be evaluated in this article, or claim that may be made by its manufacturer, is not guaranteed or endorsed by the publisher.
